# Trends in One-Year Outcomes of Dialysis-Requiring Acute Kidney Injury in Denmark 2005-2012: A Population-Based Nationwide Study

**DOI:** 10.1371/journal.pone.0159944

**Published:** 2016-07-26

**Authors:** Nicholas Carlson, Kristine Hommel, Jonas Bjerring Olesen, Anne-Merete Soja, Tina Vilsbøll, Anne-Lise Kamper, Christian Torp-Pedersen, Gunnar Gislason

**Affiliations:** 1 Department of Cardiology, Gentofte Hospital, University of Copenhagen, Gentofte, Denmark; 2 Department of Nephrology, Herlev Hospital, University of Copenhagen, Herlev, Denmark; 3 Department of Cardiology, Hvidovre Hospital, University of Copenhagen, Hvidovre, Denmark; 4 Center for Diabetes Research, Gentofte Hospital, University of Copenhagen, Gentofte, Denmark; 5 Department of Nephrology, Rigshospitalet, University of Copenhagen, Copenhagen, Denmark; 6 Institute of Health, Science and Technology, Aalborg University, Aalborg, Denmark; Postgraduate Medical Institute, INDIA

## Abstract

**Background:**

Dialysis-requiring acute kidney injury (AKI) is associated with substantial mortality and risk of end-stage renal disease (ESRD). Despite considerable growth in incidence of severe AKI, information pertaining to trends in outcomes remains limited. We evaluated time trends in one year risks of ESRD and death in patients with dialysis-requiring AKI over an eight year period in Denmark.

**Methods:**

In a retrospective nationwide study based on national registers, all adults requiring acute renal replacement therapy between 2005 and 2012 were identified. Patients with preceding ESRD were excluded. Through individual-level cross-referencing of administrative registries, information pertaining to comorbidity, preceding surgical interventions, and concurrent other organ failure and sepsis was ascertained. Comparisons of period-specific one year odds ratios for ESRD and death were calculated in a multiple logistic regression model.

**Results:**

A total of 13,819 patients with dialysis-requiring AKI were included in the study. Within one year, 1,017 (7.4%) patients were registered with ESRD, and 7,908 (57.2%) patients died. The one-year rate of ESRD decreased from 9.0% between 2005 and 2006 to 6.1% between 2011 and 2012. Simultaneously, the one-year mortality rate decreased from 58.2% between 2005 and 2006 to 57.5% between 2011 and 2012. Consequently, the adjusted odds ratios for the period 2011–2012 (with the period 2005–2006 as reference) were 0.75 (0.60–0.95, p = 0.015) and 0.87 (95% CI 0.78–0.97, p = 0.010) for ESRD and death, respectively.

**Conclusions:**

In a nationwide retrospective study on time trends in one year outcomes following dialysis-requiring AKI, risk of all-cause mortality and ESRD decreased over a period of 8 years.

## Introduction

Acute kidney injury (AKI) continues to be a severe disease associated with poor prognosis [1, 2]. AKI is independently associated with diminished short- and long-term survival [3–5], and outcomes are proportional to AKI severity [6, 7]. Additionally, AKI is associated with substantially increased propensity for chronic kidney disease (CKD) including end-stage renal disease (ESRD) [8–11]. Crude incidence rates of dialysis-requiring AKI have increased substantially throughout the last two decades [12, 13]; however, Danish incidence rates have remained stable at approximately 350 per million since 2006 [14], and information pertaining to temporal change in outcomes following AKI continues to be limited [15, 16]. Development of AKI is predominantly characterized by concurrence of acute and chronic disease [17, 18]. Consequently, risk prediction in AKI is dependent on an accurate comprehension of the interplay between potential risk factors for adverse outcomes. The clinical implications associated with changing employment of acute renal replacement therapy (RRT) however remain undetermined. Accordingly, the aim of the present study was to investigate temporal change in outcomes following dialysis-requiring AKI. Consequently, we assessed one-year risk for ESRD and all-cause mortality following dialysis-requiring AKI between 2005 and 2012 in a nationwide population-based cohort.

## Methods

### Data sources

Danish residents are issued a civil registration number at birth or time of immigration. As health services are tax-funded; all Danish citizens receive comprehensive medical coverage through the public health care system. Treatment is registered according to the civil registration number, and consequently, cross-referencing of registers is possible. Information concerning public health care is recorded in multiple national administrative registers. All hospitalizations are recorded in the National Patient Registry, and reimbursement is contingent on accurate registration by departments. Diagnostic coding is registered in accordance with the 10^th^ edition of the International Classification of Diseases (ICD), and procedural coding is registered in accordance with the Nordic Medico-Statistical Committee Classification of Surgical Procedures [19, 20]. The predicative value of diagnostic codes included in the Charlson Index Score has recently been validated with excellent results [21]. Additionally, chronic dialysis treatment and renal transplantations are recorded in the validated Danish National Registry on Regular Dialysis and Transplantation [22]. Dispensation of prescription medication by Danish pharmacies is recorded in the Danish Register of Medicinal Product Statistics[23]. The register records the Anatomical Therapeutic Chemical Classification System (ATC) code, quantity, strength and date of all prescriptions dispensed in Danish pharmacies. Prescription medication is partially reimbursed by the public healthcare system, and the register has been validated with excellent results [23, 24]. Limited biochemistry was available for a fraction of the study population; results were accessible from multiple laboratories across Denmark. Finally, information relating mortality and causes of death was determined via the National Registry of Causes of Death [25].

### Study Population

**Fig 1:**
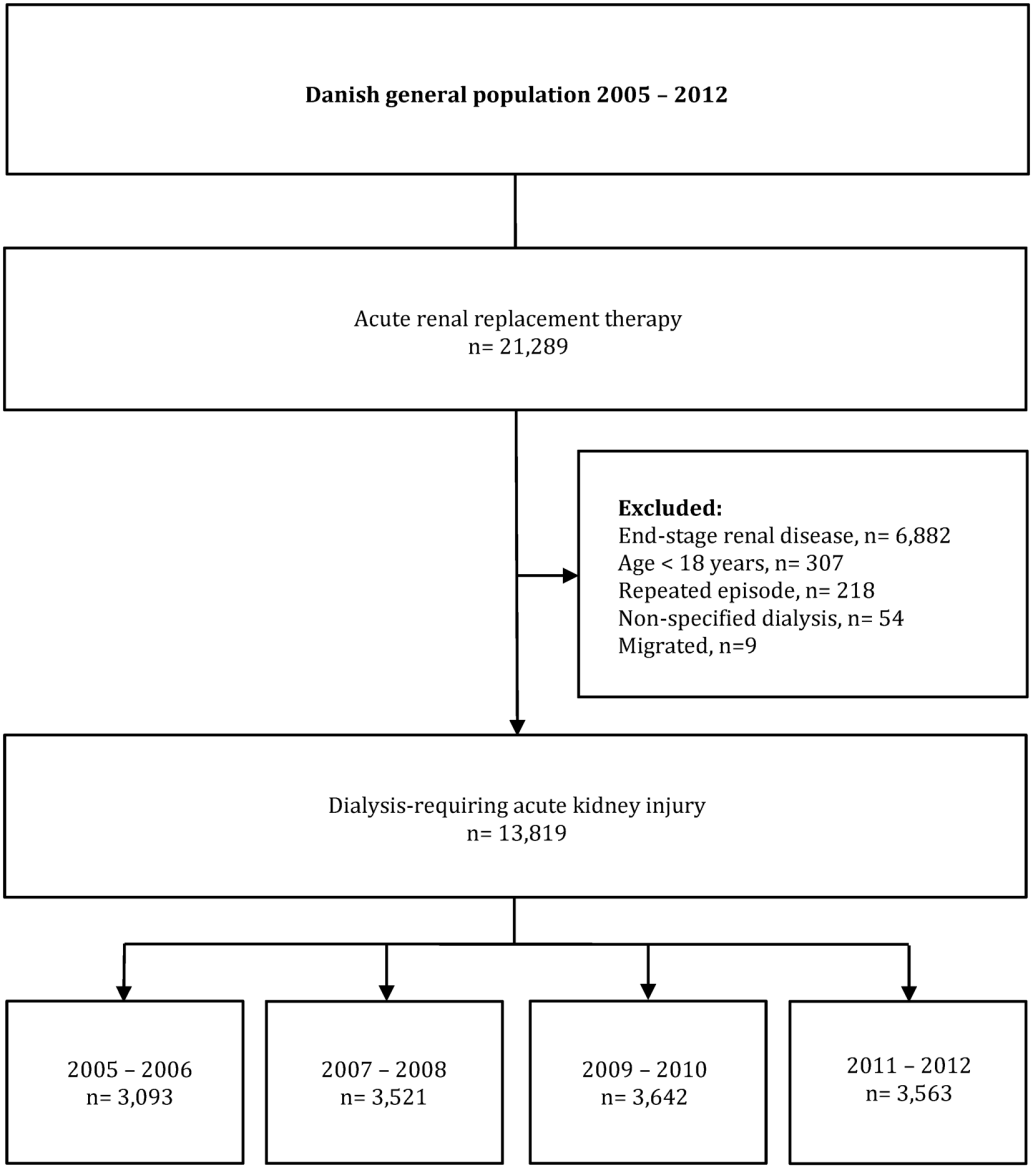
Flow chart depicting study design.

Dialysis-requiring AKI was defined as treatment with acute RRT in patients without previously registered chronic RRT. All patients requiring acute RRT between 1^st^ January 2005 and 31^st^ December 2012 were identified in the Danish National Patient Registry. Patients <18 years old, patients migrating within one year of treatment, patients previously treated with acute RRT, and patients previously registered with chronic RRT were excluded. All patients were followed until an endpoint or the end of the study. A flow chart depicting study design is shown in Fig 1.

### Study Outcomes

Primary outcomes were defined as all-cause mortality and ESRD at one year. Secondary outcomes were defined as estimated glomerular filtration rate (eGFR) (plasma creatinine measurements recorded 52–104 weeks following index) in survivors with preserved renal function at one year, duration of dialysis-requirement, and duration of hospital admission.

### Study Covariates

Comorbidities were identified based on diagnostic codes found in the National Patient Registry. Sensitivity for hypertension, and diabetes was augmented through the inclusion of information pertaining to outpatient medication. Preceding eGFRs were calculated as means all available plasma creatinine measurements recorded 52 to 2 weeks prior to index in accordance with the Chronic Kidney Disease Epidemiology Collaboration equation [26]. Azotemic parameters registered one day prior to index were included where available. Major cardiothoracic, gastric and orthopedic surgical interventions were identified based on procedural codes found in the National Patient Registry. Post-procedural dialysis-requirement was defined as acute RRT within 14 days of a major surgical intervention. Dialysis modalities were identified based on procedural codes found in the National Patient Registry as continuous RRT (CRRT), acute intermittent hemodialysis, or acute peritoneal dialysis. Similarly, requirements of mechanical ventilation and/or circulatory support were also identified based on procedural codes found in the National Patient Registry. Outpatient medications as baseline were identified in the prescription database. A comprehensive list of the medications, relevant codes, and algorithms are provided in S1 Appendix.

### Statistical Analysis

Outcomes following dialysis-requiring AKI were compared between the periods; 2005–2006, 2007–2008, 2009–2010, and 2011–2012. Trends in patient characteristics were compared using the Cochrane-Armitage test. Durations of dialysis-requirement and hospital stay amongst patients surviving beyond hospital discharge were calculated in whole days in renal survivors. Statistical significance was defined as a two sided p-value <0.05. Analyses were performed using SAS software (versions 9.4, SAS Institute), and R version 2.15.2 (R Development Core Team). Summary results were reported with 95% confidence intervals (CI), means with standard deviations (SD), and medians with interquartile range (IQR). Odds ratios for one-year mortality and ESRD were calculated in multiple logistic regression analyses with adjustment for known risk factors associated with prognosis. As such, the models were adjusted for patient age, gender, dialysis modality, comorbidity, surgery, sepsis, mechanical ventilation, and circulatory support. Comorbidities included in the model were; non-insulin dependent and insulin-dependent diabetes, congestive heart failure, ischemic heart disease, cardiac arrhythmia, valvular heart disease, peripheral vascular disease, stroke, chronic obstructive pulmonary disease, solid and non-solid cancer, and chronic kidney disease. Preceding surgeries included in the model were any major cardiothoracic, gastric or orthopedic procedures.

Furthermore, odds ratios for one-year mortality and ESRD were also computed in various selected subpopulations. These included; gender-specific strata, age-specific strata, modality-specific strata, non-surgery-related and surgery-related strata, non-intensive care requiring and intensive care requiring strata, and Charlson Index Score stratified (<3, 3–6, and >6). In additional analyses, odds ratios were calculated for 90-day mortality and 90-day renal survival using multiple logistic regression analyses analogous to the principal analyses. As incidence of dialysis-requiring AKI in the general population was lowest in 2005, sensitivity analyses with exclusion of patients from the aforementioned year were performed to confirm the principal results; no differences were discernable. Finally, residual renal function was assessed in renal survivors by comparing the proportionate number of patients with significant (>20%) decrease in eGFR 90–365 days following index. Retrospective register based studies do not need ethical approval in Denmark; however, the Danish Data Protection Agency has approved use of data (ref. 2007-58-015 / I-suite nr 00916 GEH-2010-001).

## Results

A total of 13,819 patients with dialysis-requiring AKI were identified between January 1^st^ 2005 and December 31^st^ 2012. Although patient characteristics principally remained stable, use of CRRT and mechanical ventilation increased over time. Period-specific baseline characteristics are shown in Table 1.

**Table 1:** Period-specific baseline characteristics of patients with dialysis-requiring AKI in Denmark between 2005 and 2012.

Variables	2005–2006	2007–2008	2009–2010	2011–2012	
	n = 3.093	n = 3,521	n = 3,642	n = 3.563	p-value
Characteristics					
Gender (male), n (%)	2022 (65.4)	2164 (61.5)	2264 (62.2)	2227 (62.5)	0.007
Age (years) median [IQR]	69.0 [59.2–76.3]	68.1 [58.6–76.0]	68.6 [59.3–76.3]	69.0 [60.0–76.9]	0.002
Comorbidities					
Cardiac arrhythmia (%)	691 (22.3)	720 (20.4)	813 (22.3)	867 (24.3)	0.002
Chronic kidney disease, n (%)	827 (26.7)	900 (25.6)	892 (24.5)	876 (24.6)	0.126
Chronic obstructive pulmonary disease, n (%)	339 (11.0)	385 (10.9)	375 (10.3)	425 (11.9)	0.174
Diabetes, n (%)	667 (21.6)	835 (23.7)	955 (26.2)	977 (27.4)	<0.001
Heart failure, n (%)	496 (16.0)	438 (12.4)	481 (13.2)	512 (14.4)	<0.001
Ischemic heart disease, n (%)	708 (22.9)	653 (18.5)	696 (19.1)	681 (19.1)	<0.001
Liver disease, n (%)	247 (8.0)	325 (9.2)	350 (9.6)	344 (9.7)	0.069
Non-solid cancer (%)	166 (5.4)	196 (5.6)	225 (6.2)	208 (5.8)	0.506
Peripheral vascular disease, n (%)	369 (11.9)	401 (11.4)	365 (10.0)	359 (10.1)	0.022
Solid cancer (%)	461 (14.9)	510 (14.5)	584 (16.0)	564 (15.8)	0.217
Stroke, n (%)	456 (14.7)	517 (14.7)	517 (14.2)	500 (14.0)	0.792
Valvular heart disease (%)	331 (10.7)	346 (9.8)	405 (11.1)	429 (12.0)	0.027
Charlson Index Score					
Charlson Score <3 (%)	323 (10.4)	434 (12.3)	393 (10.8)	372 (10.4)	0.098
Charlson Score 3–6 (%)	1591 (51.4)	1790 (50.8)	1895 (52.0)	1890 (53.0)	
Charlson Score >6 (%)	1179 (38.1)	1297 (36.8)	1354 (37.2)	1301 (36.5)	
Hospital admission					
Continuous renal replacement therapy, n (%)	1499 (48.5)	1858 (52.8)	2057 (56.5)	2094 (58.8)	<0.001
Acute intermittent hemodialysis, n (%)	1576 (51.0)	1649 (46.8)	1576 (43.3)	1445 (40.6)	
Acute peritoneal dialysis, n (%)	18 (0.6)	14 (0.4)	9 (0.2)	24 (0.7)	
Admission to intensive care, n (%)	2193 (70.9)	2487 (70.6)	2665 (73.2)	2640 (74.1)	0.002
Mechanical ventilation, n (%)	2010 (65.0)	2279 (64.7)	2434 (66.8)	2395 (67.2)	0.059
Circulatory support, n (%)	851 (27.5)	964 (27.4)	945 (25.9)	868 (24.4)	0.009
Sepsis, n (%)	760 (24.6)	887 (25.2)	930 (25.5)	778 (21.8)	<0.001
Cardiac surgery (%)	447 (14.5)	370 (10.5)	434 (11.9)	479 (13.4)	<0.001
Gastric surgery (%)	485 (15.7)	532 (15.1)	482 (13.2)	503 (14.1)	0.022
Orthopedic surgery (%)	160 (5.2)	174 (4.9)	191 (5.2)	180 (5.1)	0.942
Outpatient medication					
Insulin (%)	243 (7.9)	313 (8.9)	348 (9.6)	351 (9.9)	0.025
Loop diuretics (%)	929 (30.0)	1063 (30.2)	1062 (29.2)	1113 (31.2)	0.293
Thiazides (%)	409 (13.2)	507 (14.4)	551 (15.1)	501 (14.1)	0.162
Aldosterone antagonist (%)	267 (8.6)	278 (7.9)	287 (7.9)	298 (8.4)	0.611
Renin-angiotensin system blocking treatment, n (%)	869 (28.1)	1067 (30.3)	1199 (32.9)	1191 (33.4)	<0.001
Lipid-lowering treatment, n (%)	651 (21.0)	865 (24.6)	1071 (29.4)	1077 (30.2)	<0.001
Non-steroidal anti-inflammatory drugs (%)	342 (11.1)	361 (10.3)	340 (9.3)	301 (8.4)	0.002
Proton-pump inhibitors (%)	619 (20.0)	780 (22.2)	859 (23.6)	911 (25.6)	<0.001

Preceding eGFRs were available in 16.5% (n = 2,286) of patients; period-specific eGFRs were; 55 ml/min/1.73m^2^ [IQR 33–76], 57 ml/min/1.73m^2^ [IQR 37–75], 60 ml/min/1.73m^2^ [IQR 41–81], and 66 ml/min/1.73m^2^ [IQR 42–86] for the periods 2005–2006, 2007–2008, 2009–2010, and 2011–2012, respectively (p<0.001). The median time from admission to initiation of dialysis decreased; the period-specific intervals were 6 [IQR 1–18], 5 [1–14], 5 [1–14], and 4 [1–13] for the periods 2005–2006, 2007–2008, 2009–2010, and 2011–2012, respectively (p<0.001). Additionally, blood samples pertaining to dialysis indications were available in a limited number of patients at index; results are shown in Table 2.

**Table 2:** Period-specific baseline azotemia and lactate parameters.

Variables	n	2005–2006	2007–2008	2009–2010	2011–2012	p-value
Serum creatinine (μmol/L), median [IQR]	1,553	346 [188–559]	338 [183–565]	319 [156–562]	378 [188–595]	0.408
Serum K^+^ (mEq/L), median [IQR]	1,550	4.3 [3.7–4.9]	4.2 [3.7–4.8]	4.3 [3.8–4.9]	4.2 [3.6–4.9]	0.301
Serum HCO3^-^ (mEq/L), median [IQR]	287	17.1 [15.3–20.4]	18.3 [14.9–2.6]	16.1 [12.9–19.5]	16.5 [13.4–19.9]	0.056
Serum Lactate (mEq/L), median [IQR]	165	2.0 L [1.1–2.0]	1.6 [1.2–2.3]	2.2 [1.3–6.1]	2.5 [1.4–4.9]	0.048

Within one year of dialysis-requiring AKI, 1,017 patients were registered with ESRD, and 7,908 patients died, corresponding to an absolute one-year rate of 7.4% and 57.2% for ESRD and death, respectively. Prevalence of ESRD in renal survivors only was 17.2%. Period-specific unadjusted one-year mortality rates were 58.2% (n = 1,799), 56.3% (n = 1,981), 57.1% (n = 2,081), and 57.5% (2,047) for the periods 2005–2006, 2007–2008, 2009–2010, and 2011–2012, respectively (p = 0.470). Period-specific unadjusted one year rates of ESRD were 11.1% (344), 9.2% (488), 14.9% (240), and 6.6% (354) for the periods 2005–2006, 2007–2008, 2009–2010, and 2011–2012, respectively (p<0.001).

**Fig 2:**
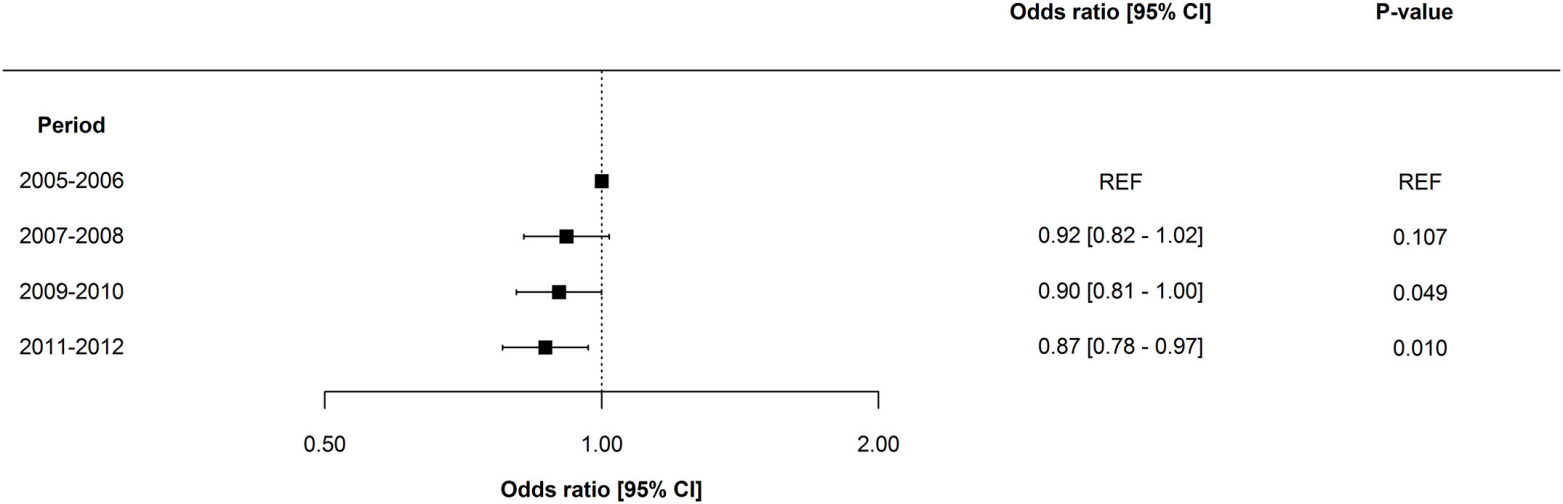
Period-specific one-year risk of death. Multivariable logistic regression model adjusted for patient age, gender, dialysis modality, comorbidity, surgery, sepsis, mechanical ventilation, and circulatory support.

**Fig 3:**
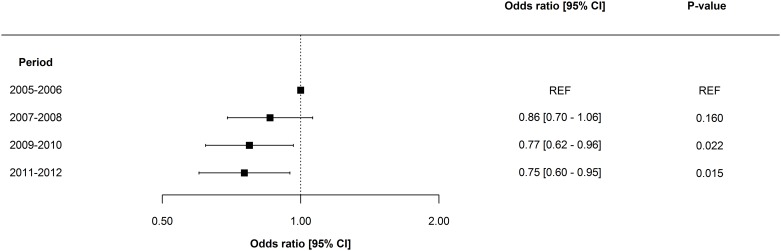
Period-specific one year risk of end-stage renal disease. Multivariable logistic regression model adjusted for patient age, gender, dialysis modality, comorbidity, surgery, sepsis, mechanical ventilation, and circulatory support.

**Fig 4:**
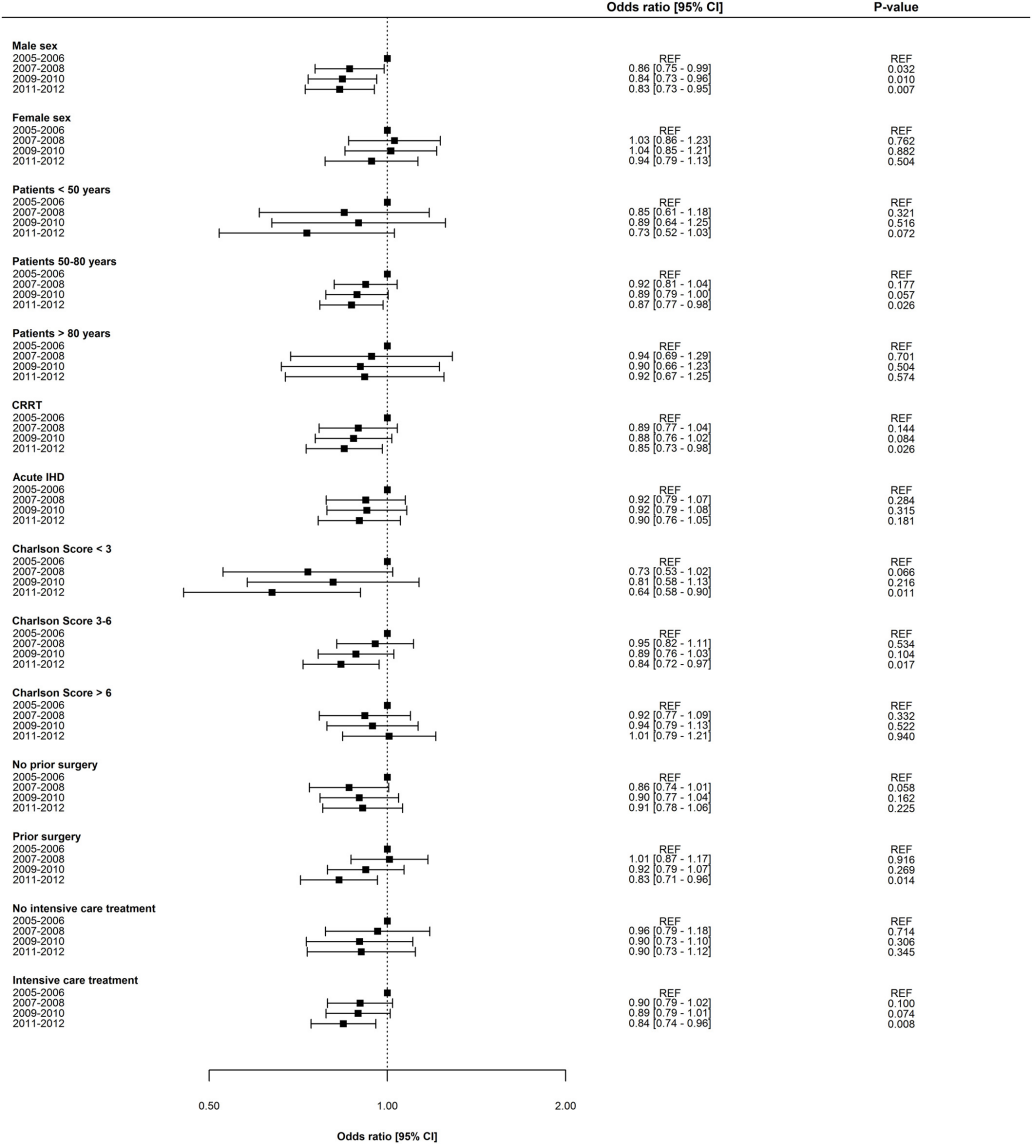
Period-specific one year risk of death in subgroups. Multivariable logistic regression model adjusted for patient age, gender, dialysis modality, comorbidity, surgery, sepsis, mechanical ventilation, and circulatory support. CRRT = Continous renal replacement therapy. IHD = Intermitted hemodialysis.

**Fig 5:**
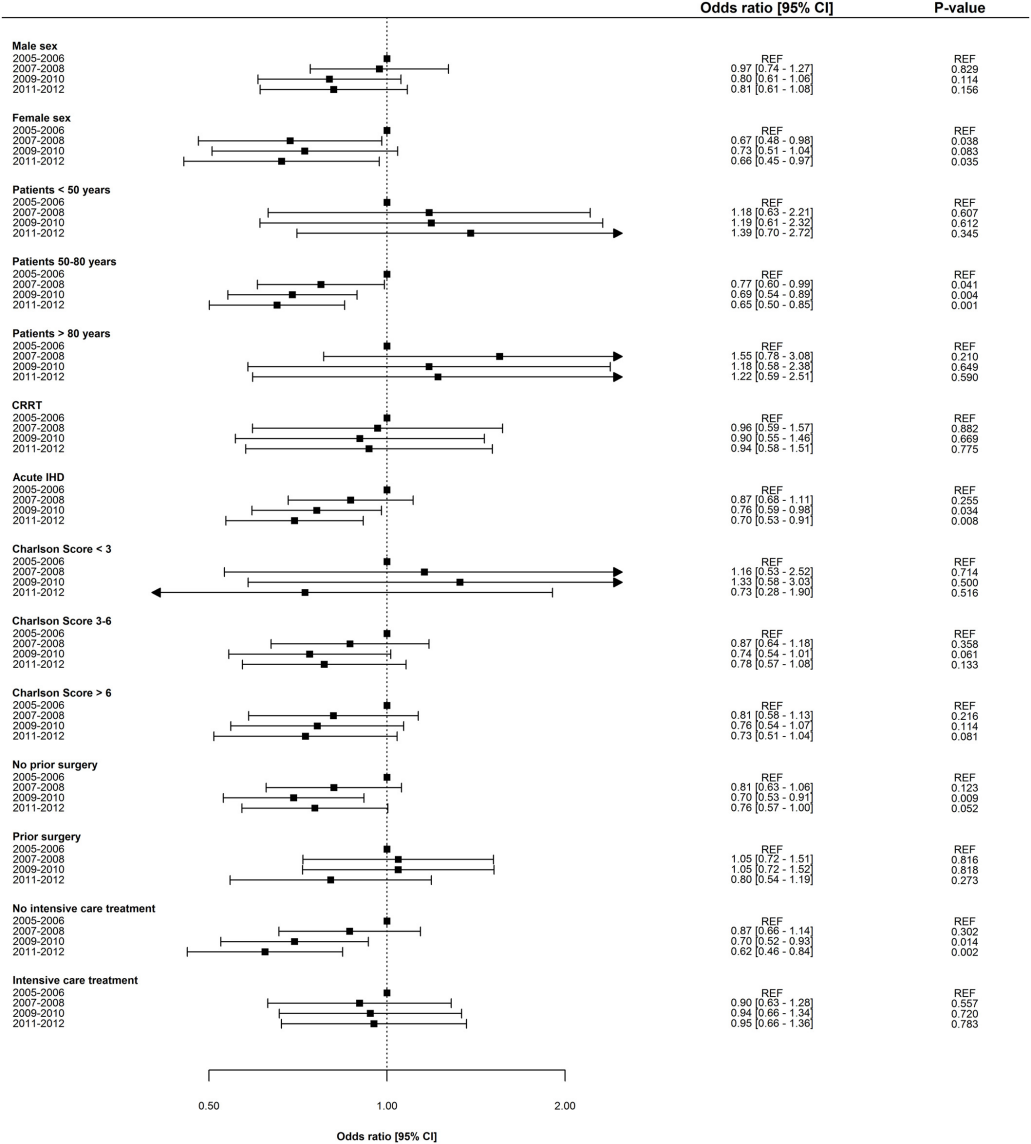
Period-specific one year risk of end-stage renal disease in subgroups. Multivariable logistic regression model adjusted for patient age, gender, dialysis modality, comorbidity, surgery, sepsis, mechanical ventilation, and circulatory support. CRRT = Continous renal replacement therapy. IHD = Intermitted hemodialysis.

Overall, adjusted odds ratios for ESRD and death decreased incrementally over time. The adjusted one-year risk of death decreased by 3.8% [2.1–5.4] per year (p = 0.001), and the adjusted one-year risk of ESRD decreased by 6.6% [3.3–9.8] per year (p = 0.009). Odds ratios for one-year risk of death and ESRD are shown in Figs 2 and 3, respectively. Notably, mortality remained essentially unchanged in women, patients >80years, and in patients with Charlson Index score >6; however, ESRD prognosis for all subpopulations improved in all subpopulations. No significant interaction was determined between period and gender, period and patient age, and period and dialysis modality. Odds ratios for one-year risk of death and ESRD in subpopulations are shown in Figs 4 and 5. Improvement in short-term risk of death and ESRD was also observed. Odds ratios for 90-day mortality were 0.90 (0.81–1.00), 0.94 (0.84–1.04), and 0.86 (0.77–0.96), and odds ratios for 90-day risk of ESRD were 0.92 (0.75–1.12), 0.81 (0.66–1.00), and 0.76 (0.61–0.95) for the periods 2007–2008, 2009–2010, and 2011–2012, respectively (with 2005–2006 as reference).

Preceding and succeeding eGFRs were available in a total of 493 of 5,911 renal survivors. Overall, a ≥20% decreased in eGFR following dialysis-requiring AKI was observed in 43.2% (213) patients in the subsample; the period-specific proportions of patients with a ≥20% decrease in eGFR were 51.2%, 41.1%, 38.2%, and 43.6% for the periods 2005–2006, 2007–2008, 2009–2010, and 2011–2012, respectively (p = 0.125). Durations of dialysis-requirement decreased over time; the period-specific median requirements were 13 days [IQR 6–25], 12 days [IQR 5–24], 12 days [IQR 6–23], and 12 days [IQR 6–22], for the periods 2005–2006, 2007–2008, 2009–2010, and 2011–2012, respectively (p<0.001). Durations of hospital admission also decreased over time; the period-specific median requirements were 70 days [IQR 34–111], 63 days [IQR 24–104], 63 days [IQR 30–108], and 54 days [IQR 26–96], for the periods 2005–2006, 2007–2008, 2009–2010, and 2011–2012, respectively (p<0.001).

## Discussion

In this nationwide retrospective study on outcomes following dialysis-requiring AKI, prognosis was observed to improve incrementally during an eight year study period. Specifically, the odds ratio of both all-cause mortality and ESRD was observed to decrease by ~20% between 2005 and 2012. Simultaneously, durations of dialysis-requirement and hospitalization decreased. Irrespectively, prognosis following dialysis-requiring AKI remains unfavorable, with only approximately one third of patients surviving without requiring chronic RRT. AKI is associated with significant mortality, with one-year mortality rates fluctuating from ~35% to ~70% dependent on population and AKI severity [4, 27–29]. However, mortality rates have been reported to be decreasing [13, 15, 16, 30, 31], although the attributable risk for death associated with AKI may be increasing [32]. Additionally, AKI survivors remain at increased risk of ESRD. Reported rates of ESRD range from 2% to 20% [3, 5, 8, 15, 33, 34]; however, risk of ESRD following dialysis-requiring AKI—as is the case with mortality—could be decreasing [15]. Regardless, our results indicate incremental improvement in prognosis following dialysis-requiring AKI from 2005 till 2012.

Although initiation of acute RRT in the context of imminent indications is associated with poorer outcomes [35], evidence supporting early initiation of acute RRT in AKI remains wholly insufficient [36]. Accordingly, initiation of acute RRT continues to be based on imminent indications with no apparent shift towards earlier initiation of therapy [37, 38]. Our results corroborate this observation, as we were unable to document any substantial change in pre-dialysis azotemia or serum lactate. Furthermore, incidence rates of non-dialysis-requiring AKI have increased throughout the last decade, while incidence rates of dialysis-requiring AKI have remained stable [14]. As such, no existing evidence clearly substantiates changing thresholds for initiation of acute RRT.

Although no novel interventions pertaining to dialysis-requiring AKI have demonstrated genuine benefit throughout the last decade [39–43], our results nonetheless provide further evidence of increments in survival and renal recovery following severe AKI. Use of CRRT increased throughout the study period. Dialysis-modality has previously been demonstrated to be associated with outcomes in observational data [38]; however, the association is plausibly driven by selection biases, and no evidence currently supports any benefit associated with a specific modality [44–48]. Improvements in prognoses could alternately represent the beneficial effects related to implementation of evidence-based goal-directed therapy in critical illness [49, 50]. Outcomes following sepsis and septic shock are known to be improving [51, 52], possibly due to the benefits associated with the surviving sepsis guidelines [53], and outcomes following acute respiratory failure may also be improving [54]; albeit, thresholds for use of mechanical ventilation could be changing [55]. As such, the observed improvement of outcomes following dialysis-requiring AKI remains plausible, although possibly driven by interventions targeting supportive and non-kidney related care.

Our results confirm the detrimental consequences associated with severe AKI. Initial survivors of dialysis-requiring AKI remain at substantially increased risk of ESRD. Tentatively, our results indicate an improvement in residual eGFR in non-ESRD survivors; however, as preceding and succeeding eGFRs were only available in a small fraction of our population, the analysis was both underpowered and plausibly subject to certain biases. While outpatient care holds potential in identifying CKD in time to mitigate complications, only a fraction of patients with dialysis-requiring AKI are currently referred to a nephrologist following discharge [56, 57]. Notably, referral to a nephrologist following discharge is associated with interventions [58], and improved outcomes in retrospective cohorts [59].

Our study had numerous strengths. First, due to the structure of public health care in Denmark, national registries record comprehensive information pertaining to medical care of all Danish citizens. Follow-up is generous and essentially unflawed, and national registries are extensively validated. Second, the availability of reliable data from the Danish National Registry on Regular Dialysis and Transplantation ensures an accurate dissemination between dialysis-requiring AKI and acute RRT in ESRD. Additionally, the accuracy of CRRT in the intensive care setting has previously been validated with excellent results [60]. However, our study also had a number of limitations. First, correlation is not causation; due to the observational design, our results do not provide definite answers related to cause. Second, the absence of systematic and universal laboratory and clinical data is regrettable; consequently, a number of possible confounders remain unaddressed. Specifically, the limited data on biochemical and clinical indications for initiating acute RRT remains unfortunate; particularly as the availability of data pertaining to baseline azotemia was underpowered for definite conclusions regarding clinical implications. Furthermore our algorithm for identification of dialysis-requiring AKI does not rule out alternate indications for acute RRT to AKI. Additionally, prevalence of comorbidity may be underestimated due to a reliance on diagnostic and procedural coding. Finally, due to the particular demographics of Denmark, results cannot necessarily be extrapolated to other populations.

### Conclusions

In a nationwide retrospective study on outcomes within the first year following dialysis-requiring AKI, we observed incremental decrease in odds ratios for all-cause mortality and ESRD between 2005 and 2012. Although mortality of patients with greatest vulnerability and women remained unchanged, an overall improvement in outcomes following dialysis-requiring AKI of all patients was observed. Although interpretation of our results is subject to limitations pertaining to residual confounding, dialysis-requiring AKI nonetheless continues to be associated with disheartening prognoses, with current one-year mortality and ESRD rates exceeding 50% and 10%, respectively.

## Supporting Information

S1 Appendix Supplementary methods. (DOCX)

## Abbreviations

AKI: Acute kidney injury
ATC: Anatomical therapeutic chemical classification system
CI: Confidence interval
CKD: Chronic kidney disease
CRRT: Continuous renal replacement therapy
eGFR: Estimated glomerular filtration rate
ESRD: End-stage renal disease
ICD: International classification of diseases
IQR: Interquartile range
RRT: Renal replacement therapy
SD: Standard deviation
