# Supplementary material for: Trends in One-Year Outcomes of Dialysis-Requiring Acute Kidney Injury in Denmark 2005-2012: A Population-Based Nationwide Study
Source: PLoS One. 2016 Jul 26;11(7):e0159944. doi: 10.1371/journal.pone.0159944 (PMC4961397; doi:10.1371/journal.pone.0159944)
Supplement: S1 Appendix — (DOCX) [file pone.0159944.s001.docx]

**Methods**

Initial dialysis modality was identified in the Danish national patient registry based on the Nordic Medico-Statistical Committee Classification of Surgical Procedures. Modalities were; acute intermittent hemodialysis (procedural code ‘BJFD00’), acute peritoneal dialysis (procedural code ‘BJFD01’), and continuous renal replacement therapy (procedural code ‘BJFD02’).

Diabetes was identified by diagnosis with ICD-10 code ‘DE10-14’ or by prescription of any anti-diabetic medication defined as ATC code ‘A10’. Heart failure was identified by ICD-10 code ‘DI50’. CKD was identified by ICD-10 codes ‘DN18’ (chronic renal insufficiency), ‘DN19’ (renal insufficiency), ‘DN391’ (chronic proteinuria), ‘DN25-DN29’ (other disorders of the kidney and ureter), ‘DN03-DN08’ (glomerular diseases) ‘DN11’ (Chronic tubulo-interstitial nephritis), ‘DN14’ (Drug- and heavy-metal-induced tubulo-interstitial and tubular conditions), ‘DE102’ and ‘DE112’ (Diabetic nephropathy), ‘DI12-13’ (hypertensive nephropathy), ‘DQ61’ (cystic kidney disease) and ‘DN06’ (proteinuria). Ischemic heart disease was identified by ICD-10 codes ‘DI20’, ‘DI21’, ‘DI22’, ‘DI23’, ‘DI24’ and ‘DI25’. Peripheral vascular disease was identified by ICD-10 codes ‘DI70’, ‘DI71’, ‘DI72’, ‘Di73’ and ‘DR02’. Ischemic or hemorrhagic stroke was identified by ICD-10 codes ‘DI61’, ‘DI62’, ‘DI63’, ‘DI64’, ‘DI691’, ‘DI692’, ‘DI693’, and ‘DI694’, Valvular heart disease was identified by any ICD-10 code between ‘DI34’ and ‘DI36’. Non-solid cancer was identified by any ICD-10 code between ‘DC81’ and ‘DC94’, and solid cancer was identified by any ICD-10 code between ‘DC00’ and ‘DC80’ (with exception of ‘DC44’). Cardiac arrhythmia was identified by any ICD-10 code between ‘DI48’ and ‘DI49’. Liver disease was identified by ICD-10 codes between ‘DK70’ to ‘DK77, and ‘DB15’ to ‘DB19’, and ‘DC22’, ‘DC944’, ‘DI982B’ and ‘DQ618A’. Chronic obstructive pulmonary disease was defined by ICD-10 codes ‘DJ42-44’.

The duration of hospitalization was calculated as the time between first admission and last discharge, and the duration of dialysis-requiring AKI was calculated as the time between first and last acute dialysis treatment. Information pertaining to other organ failure by requirement of mechanical ventilation (procedural code ‘BGD’), and/or circulatory support (procedural code ‘BFHC92’) based on records in the National Patient Registry. Correspondingly, surgical AKI was defined on the basis of surgical procedures performed within the last 14 days prior to index. Surgery was classified as cardiac (procedural code ‘KF’), gastric (procedural code ‘KJC’ to ’KJN’), orthopedic (procedural code ‘KN’).

Finally, outpatient medications were identified by ATC-code as prescriptions redeemed within the last 90 days prior to index. The identified medications were; insulin (A10A’, ‘A10B’, and ‘A10C’), lipid-lowering treatment (‘‘C10A’ – ‘C10B’), proton-pump inhibitors (‘A02B’), non-steroidal anti-inflammatory drugs (‘M01B’), RAS-blocking drugs (‘C09A’, ‘C09B’, ‘C09C’, and ‘C09D’), aldosterone antagonists (‘C03D’), loop-diuretics (‘C03CA01’), and thiazides (‘C03A’).
